# Detecting subtle subterranean movement via laser speckle imaging

**DOI:** 10.1242/jeb.247267

**Published:** 2024-11-22

**Authors:** Hosain Bagheri, Michael A. D. Goodisman, Daniel I. Goldman

**Affiliations:** ^1^ School of Biological Sciences, Georgia Tech, 310 Ferst Drive, Atlanta, GA 30332, USA; ^2^ School of Physics, Georgia Tech, 837 State Street NW, Atlanta, GA, 30332, USA

**Keywords:** Dynamic light scattering, Subterranean, Eusocial insect, Holometabolous, Fire ants

## Abstract

A diversity of organisms live within underground environments. However, visualizing subterranean behavior is challenging because of the opacity of most substrates. We demonstrate that laser speckle imaging, a non-invasive technique resolving nanometer-scale movements, facilitates quantifying biological activity in a granular medium. We monitored fire ants (*Solenopsis invicta*) at different developmental stages, burial depths (1–5 cm) and moisture fractions (0 and 0.1 by volume) in a container of 0.7 mm glass particles. Although the speckle pattern from the backscattered light precludes direct imaging of animal kinematics, analysis of integrated image differences revealed that spiking during ant movement increased with the developmental phase. Greater burial depth and saturation resulted in fewer and lower magnitude spikes. We verified that spiking correlated with movement via quasi-2D experiments. This straightforward method, involving a laser and digital camera, can be applied to laboratory and potentially field situations to gain insight into subterranean organism activities.

## Teaser

**Summary:** Laser speckle imaging non-invasively quantifies subterranean organism activity by detecting nanometer-scale movements.

## INTRODUCTION

Diverse organisms inhabit subterranean environments. A variety of annelids, arthropods, amphibians, reptiles and mammals live and operate underground (Nielsen et al., 2015; Bardgett and Van Der Putten, 2014). For example, a recent analysis (Rosenberg et al., 2023) suggests that 10^19^ individual arthropods inhabit the Earth's soil, with social insects such as termites and ants accounting for about 55% of the soil's biomass. Studying the behavior of subterranean organisms is difficult because, unlike imaging above-ground flying, swimming or running animals, monitoring the short-term (e.g. locomotion; Astley et al., 2020) and long-term (e.g. phenology; Bonato Asato et al., 2023) dynamics of organisms within substrates such as soil or leaf litter is not easily achievable. This is in part because of the challenges of imaging through optically opaque media.

Researchers ranging from geophysicists to ecologists have developed tools for examining, exploring and detecting subterranean environments or associated subterranean objects and infrastructures (Banaseka et al., 2021; Zrinjski et al., 2012). Because of the optical opacity of common substrates, such tools typically do not rely on light-based imaging but instead capitalize on acoustic (Xing et al., 2018; Liu et al., 2020) or non-visible electromagnetic waves (Kisseleff et al., 2018; Wang et al., 2022) for underground detection and tracking. For example, electromagnetic waves have been used for wireless communication and detection between above-ground and underground sensors (Yu et al., 2013) and for tracking suitably tagged biological organisms (Markham et al., 2010; Noonan et al., 2015). Studying the reflection of acoustic waves transmitted through the soil (Azfar et al., 2018) yields information about subsurface composition, structures and objects. However, such techniques are typically suitable for detecting relatively larger underground structures and objects: cables, pipelines, tunnels and cavities (Wang et al., 2022; Liu et al., 2020; Roohezamin et al., 2022; Zahari et al., 2015). X-Ray imaging has been used to monitor the movement of diverse animals (Maladen et al., 2009; Sharpe et al., 2015a) but requires relatively expensive detectors and custom containers.

**Fig. 1.**
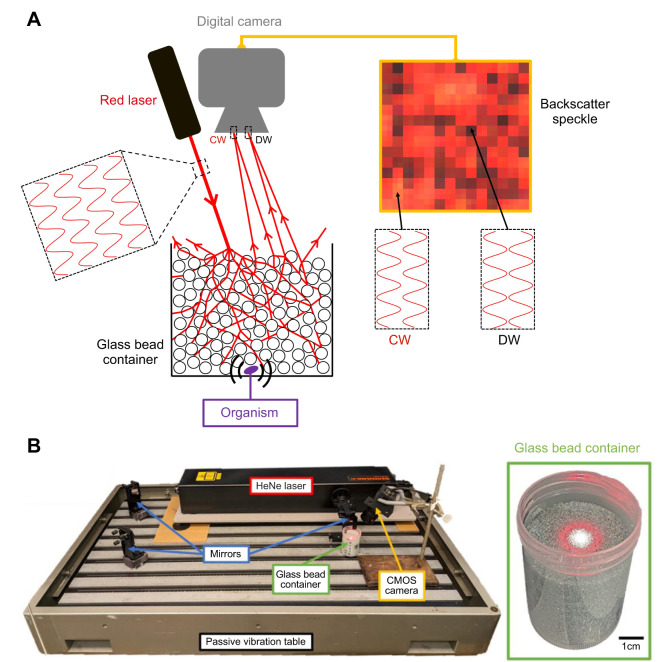
**Laser speckle imaging.** (A) Our application of laser speckle imaging (LSI) consists of focusing a laser upon a container filled with 0.7 mm glass particles and capturing the backscattered speckle pattern with a digital camera. Approximately four adjacent pixels contribute to the coherence area (a region of characteristic light or dark). CW, constructive wave interference; DW, destructive wave interference. (B) The experimental apparatus, consisting of a HeNe laser directed using mirrors onto a container filled with granular media. A CMOS camera captures the backscattered speckle pattern. Inset: the cylindrical experimental container (5 cm diameter and 8 cm height), in which ants of different developmental stages are buried under 1–5 cm of dry or saturated glass particles.

We hypothesized that a non-invasive, low-cost and minimalist technique called laser speckle imaging (LSI) could be used to detect underground activity in biological systems generating relatively small environmental deformations. LSI (Ennos, 1975; Rabal and Braga, 2018; Goodman, 1975) consists of focusing a laser, a coherent and collimated light source, on a medium and recording the scattered speckle pattern using a digital camera. As light waves transmit through the substrate, they undergo multiple scattering events. These scattered light waves give rise to an interference phenomenon characterized by the superposition of constructive and destructive waves. Constructive waves result in bright spots, while destructive waves lead to dark spots. The mean size of these bright and dark spots is known as the ‘coherence area’, representing the average spatial region where the light remains coherent (Lemieux and Durian, 1999; Goldman, 2002). If placed near the sample, a digital camera can capture the scattered light (Wolfgang, 1982), which consists of a random intensity ‘speckle pattern’ (Fig. 1A). Broadly, speckle patterns can be monitored through either transmission (where incident light scatters through the sample and is collected upon emergence) or backscatter (where scattered light penetrates the sample and reflects back to the detector positioned near the laser). In either mode, because changes in the speckle pattern result from phase shifts in light paths generated by numerous scattering events, LSI can resolve nanometer-scale deformations induced in a medium (Goldman, 2002; Weitz et al., 1992), quantified by analyzing the temporal evolution of pixel intensity within the dynamic speckle pattern.

Monitoring speckle dynamics has been widely used in medical contexts, including retinal blood flow (Fercher and Briers, 1981; Briers and Fercher, 1982, 1983; Briers, 2007; Briers et al., 2013), skin capillary perfusion (Briers and Webster, 1996; Briers, 2007) and cerebral blood flow (Cheng et al., 2003). Over time, this technique has been employed to visualize tissue perfusion in various medical fields such as ophthalmology, rheumatology, dermatology and neurology (Draijer et al., 2009; Heeman et al., 2019). However, to the best of our knowledge, the technique has not been applied to monitoring organisms within substrates. Significant theory has been developed to use the technique to extract quantitative information about the dynamics of processes in soft matter, particularly foams (Durian et al., 1991; Bandyopadhyay et al., 2005; Höhler et al., 2014), colloidal and granular dynamics (shearing; Wu et al., 1990), creeping (Pons et al., 2016; Erpelding et al., 2010), collisions (Menon and Durian, 1997) and jamming (Kim and Pak, 2010; Kim et al., 2005).

Given the length scales on which LSI operates (cm) and its ability to probe tiny subterranean deformations (nm), we posited that such a method could be valuable in probing activities of subterranean invertebrates. To this end, here we describe the development of LSI for the laboratory study of movement in dry and wet granular media. We tested the tool via monitoring entrapment in the red imported fire ant, *Solenopsis invicta* Buren 1972 (hereafter ‘fire ant’) (Tschinkel, 2013). These ants are highly invasive (Ascunce et al., 2011) and capable of creating complex nests in a variety of soils. The technique allows resolution of the time dynamics of ants of different developmental stages including minute movements associated with larval struggles. Examining different life stages should be particularly informative because of the significant variations in movement and behavior across these stages. Adult fire ants typically move multiple body lengths as they transit throughout a nest or undertake various nest maintenance activities. In contrast, the larval and pupal stages display very little mobility. For instance, larvae move their mandibles while eating, and pupae exhibit infrequent, minute contractions in the latter stages of development. Thus, the investigation of multiple life stages provides insight into the ability of this technique to detect different levels of animal activities.

## MATERIALS AND METHODS

We constructed a system to test the utility of LSI to detect subterranean motion in a model opaque substrate. On a passive vibration table, a series of four mirrors redirected a HeNe laser (Renishaw RL633, 633 nm, 17 mW) beam upon the center of the experimental container consisting of ∼700 µm glass particles. Glass particles were selected for their individual particle optical transparency, and their size and shape uniformity; the chosen size prevents animal escape through excavation while allowing movement of the particles around them. Larger and/or more opaque particles, such as natural sand, reduce light transmission as a result of their optical opacity and the increased individual particle scattering. However, even in such substrates, the technique effectively captures subterranean activity as long as sufficient particle movement occurs within the scattered light field. In LSI, scattered light can be collected at a single point via a photomultiplier. In recent years, the advent of low-cost digital cameras has allowed for convenient ‘multispeckle’ imaging (Goldman, 2002). In our apparatus, a CMOS camera (Point Grey Flea3, FL3-U3-32S2C-CS) with an attached lens (Tamron M12VM412, 4–12 mm focal length, Flat-Field Mega-Pixel Lens) recorded the backscatter for 2–10 min at 60 frames s^−1^ (Fig. 1B). In multispeckle LSI, the scattered light is directly incident on the camera's detector, typically without the use of a lens. In our case, a lens was required to position the camera near the sample to collect sufficient backscattered light from a small region at the center of the incident beam (Weitz et al., 1993). Typically, each speckle spot (a coherence area) should be comparable in dimensions to the pixel dimensions (Briers, 2007). If the speckle spot is larger than the pixel size, fewer speckles are sampled per pixel, leading to unreliable statistics. Conversely, if the speckle spot is smaller than the pixel size, each pixel becomes an average of many speckle spots, diminishing the effectiveness of the technique. In our experiments, the coherence area was about four adjacent pixels (2×2 pixels).

While, in principle, one can conduct LSI using a low-cost laser, initial tests revealed that the intensity fluctuated (Fig. S1), leading to challenges in speckle pattern interpretation. Therefore, we chose a commercial HeNe laser with high spectral stability and narrow line width. The wavelength of the laser is a critical parameter that determines its interactions with matter and needs to be tailored per application. While a shorter wavelength laser could provide finer speckle patterns, we aimed for greater penetration depth into the sample, leading us to use a longer wavelength laser. The brightness of a laser is determined by its wavelength and power density. A high-brightness laser can achieve a finer beam spot size (smaller coherence area), longer depth of field (higher spatial coherence) and increased beam stability (Shukla et al., 2015). However, excessive laser power can induce undesirable heating, which can even lead to particle displacement (Dayal and Gambaryan-Roisman, 2017; Marston and Pacheco-Vazquez, 2019).

To assess the technique's ability to detect subtle animal underground movement, we separately buried fire ants at various developmental stages under the medium at different water fractions (Sharpe et al., 2015b; Monaenkova et al., 2015) and depths. The parameters varied included: water content of the medium (0 and 0.1 by volume), ant burial depth (1, 3 and 5 cm) and ant development stage (sexual larvae, sexual unpigmented pupae, sexual pigmented pupae and worker adult). The environmental parameters were intended to model aspects of natural soils.

We used both a quasi-2D and a 3D experimental container. 3D experimental conditions consisted of burying ants along the central axis of a cylindrical container (with dimensions of 5 cm diameter and 8 cm length) (Fig. 1B). The selection of the 3D experimental container size aimed to minimize boundary effects (Stone et al., 2004; Kang et al., 2018). We performed a 2 min backscatter recording of seven individually buried ants at each development stage. These experiments are analogous to natural conditions, where the viewer possesses no visual information of the potential underground ant activities other than those associated with the speckle pattern.

**Fig. 2.**
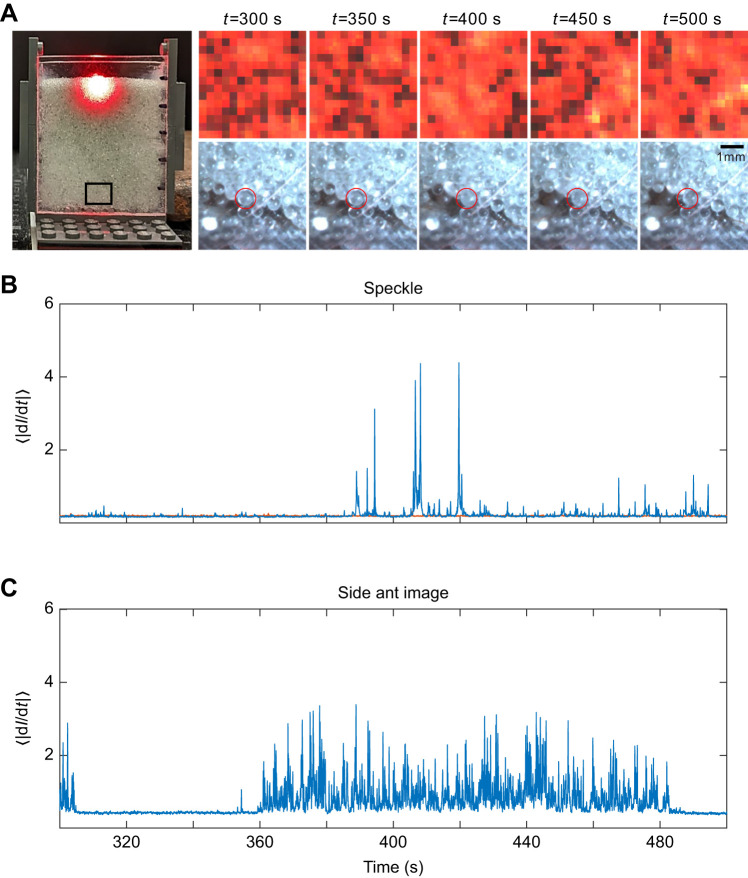
**Calibrating the LSI method via a quasi-2D setup.** (A) An experimental container (5 cm width, 6 cm height and 0.5 cm depth), showing the last third of a 10 min (i.e. from 300 to 500 s) recording of the top backscattered speckle pattern and side particle (circled in red) movement caused by a worker adult ant buried under 5 cm of dry glass particle medium. Plots show the mean absolute change of pixel intensity with respect to time, denoted as ⟨|d*I*/d*t*|⟩, for the (B) top speckle pattern and (C) side particle movement. In the resulting plots, blue signifies ant activity, while orange represents the control, denoting the absence of a buried ant.

Quasi-2D experiments enabled the concurrent observation of the laser speckle pattern from a top view and the animal's movement from the side view. This setup validated the LSI method by correlating changes in animal movement with alterations in the speckle pattern. In the quasi-2D experiments, an adult worker ant was buried within the granular medium at the center of a clear rectangular prism (with dimensions of 5 cm width, 6 cm height and 0.5 cm thickness) and recorded for 10 min (Fig. 2A).

In much of the analysis of LSI for soft matter systems, such as colloids, one typically deals with dynamics in a steady fluctuational state and no steady spatial structure, where transient dynamics can be resolved by multispeckle techniques (Höhler et al., 2014). In a ‘steady fluctuational state’, fluctuations occur consistently over time, maintaining constant statistical properties. In both cases, one examines the temporal correlation of a local signal (either photodetector or pixel) and this can be linked to aspects of the sample's dynamics (e.g. velocity distribution) (Kim and Pak, 2010; Goldman, 2002). However, because our sample contains a localized animal and the dynamics are not necessarily statistically stationary (i.e. system dynamics remain constant over time), we adopted a simpler analysis technique. The presented technique, which examines temporal changes in pixel intensity, not only detects dynamic events but also illustrates the system's evolution over time.

We analyzed the backscattered speckle pattern images by first converting the image sequence to grayscale. To mitigate noise from environmental and laser instabilities (e.g. mechanical vibrations, electrical noise, current and thermal fluctuations), we applied a 10th-order lowpass Butterworth filter with a cutoff frequency of 6 Hz, applied individually to each pixel across the temporal domain. Next, we integrated the image differences by calculating the absolute values of pixel intensity differences between each consecutive image along the temporal domain. Lastly, we computed the spatial average for each new (absolute difference) image, resulting in single positive point value per image.

We denote this metric as ⟨|d*I*/d*t*|⟩, the mean absolute change of pixel intensity with respect to time (Goldman, 2002). Implementing this protocol on backscatter recordings in the absence (control) and presence of buried ants, with animals buried at sufficient depth, allowed us to distinguish particle movement induced by ant movement. In the absence of ant movement, the speckle pattern exhibits a relatively stable state, resulting in a ⟨|d*I*/d*t*|⟩ plot characterized by minimal variations and low magnitudes. However, with ant movement, the speckle pattern undergoes noticeable changes, manifested as an increase in magnitude with varying values on the ⟨|d*I*/d*t*|⟩ plot (Fig. 2B).

Finally, we analyzed the speckle patterns in the spatial domain to determine the ability of LSI to identify the location of the buried subject. We posited that for deeply buried animals, the laser light would be sufficiently diffusive that spatial information would be lost (Höhler et al., 2014). An image generated by integrating each pixel of the speckle pattern in time should display a statistically uniform profile. For shallowly buried animals, some light could be ballistically backscattered, and the time-integrated speckle pattern should show spatial heterogeneity. To determine whether the speckle pattern contained spatial information, we computed the sum of the absolute change in pixel intensity over time (Σ|d*I*/d*t*) for each pixel within the recorded image. This yields a color map of the recorded image over time. The brighter regions of the image indicate areas with more pronounced change in pixel intensity. In contrast, the dimmer regions of the image indicate areas with less change in pixel intensity.

## RESULTS AND DISCUSSION

Using the quasi-2D apparatus, we first demonstrate that LSI can be used to monitor movements of buried animals (Fig. 2; Movie 1). In the absence of a buried ant or ant activity, the backscattered speckle pattern exhibited minimal variation in ⟨|d*I*/d*t*|⟩ (Fig. 2B). During animal movement, the particles within the scattered light field rearranged sufficiently (although not visibly) to generate changes in speckle. These rearrangements correlated with visible rearrangements of particles near the ant (monitored by calculating ⟨|d*I*/d*t*|⟩ on the visible light images), several centimeters away from the edge of the scattered light field (Fig. 2C), which spans about 1 cm. Our controls (without a buried animal; Fig. 2B, orange plot) showed minimal change in pixel intensity over time, attributed to noise within the laser and imaging system. On occasion, spikes in the speckle pattern appeared without discernible movement in the visible images. These can be related to the complex time dependence of microscopic rearrangements throughout a granular medium subjected to localized disturbances.

We also tested our method in natural dry sand consisting of semi-transparent grains. Quasi-2D experiments with a worker adult ant buried under 1, 3 and 5 cm of ∼500 µm (mean±s.d., 480±160 µm) polydisperse opaque grains with complex shapes (Fig. S2, Movie 2) revealed minimal fluctuations in the speckle field at 3 and 5 cm depths, despite large fluctuations in the visible image signal. Fluctuations at 3 cm were slightly more pronounced than those at 5 cm, while at 1 cm, the speckle field exhibited significant fluctuations. We interpret this as a result of the frictional and jagged grains' ability to resist perturbations far from the light field. In addition, the opaque medium decreased the amount of penetration of the laser field into the medium, resulting in decreased sensitivity to disturbances far from the light field. Spikes in the speckle field that were visible could occur from complex dynamics of the non-visible settling granular medium (on micrometer scales) due to inadvertent mechanical agitation or temperature fluctuations.

Confident that we could resolve animal activity sufficiently far from the scattered light field, we next analyzed the speckle patterns from the 3D setup for fire ants at the four distinct developmental stages and different burial depths. The technique detected activity in all cases, shown as noisy spiking time series of ⟨|d*I*/d*t*|⟩.

**Fig. 3.**
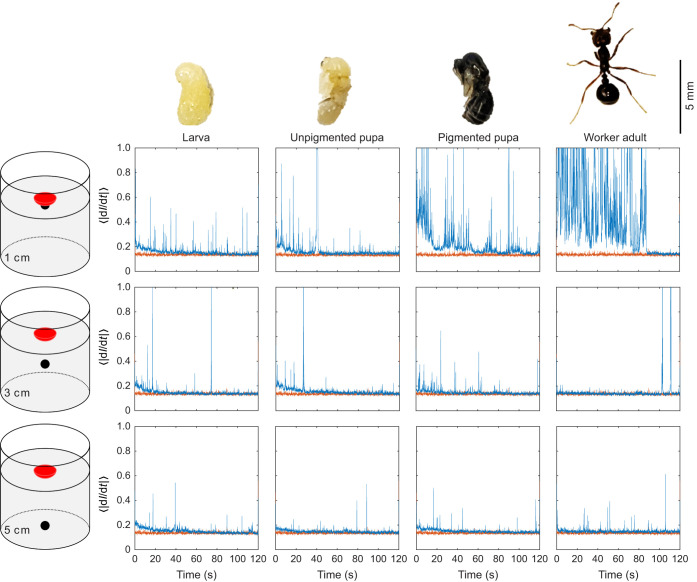
**LSI reveals activity of fire ants at various life stages.** The life stages shown are larva (L), unpigmented pupa (UP), pigmented pupa (PP) and worker adult (WA). Specimens were placed under dry glass particles at depths of 1, 3 and 5 cm within a cylindrical container (5 cm diameter and 8 cm length). In the resulting plots, blue/orange corresponds to the presence/absence of a buried animal. Mean activity, defined by the average ⟨|d*I*/d*t*|⟩, was as follows: control 0.14, L_1_ 0.16, L_3_ 0.15, L_5_ 0.15, UP_1_ 0.18, UP_3_ 0.16, UP_5_ 0.15, PP_1_ 0.23, PP_3_ 0.15, PP_5_ 0.15, WA_1_ 0.53, WA_3_ 0.15, WA_5_ 0.15.

**Fig. 4.**
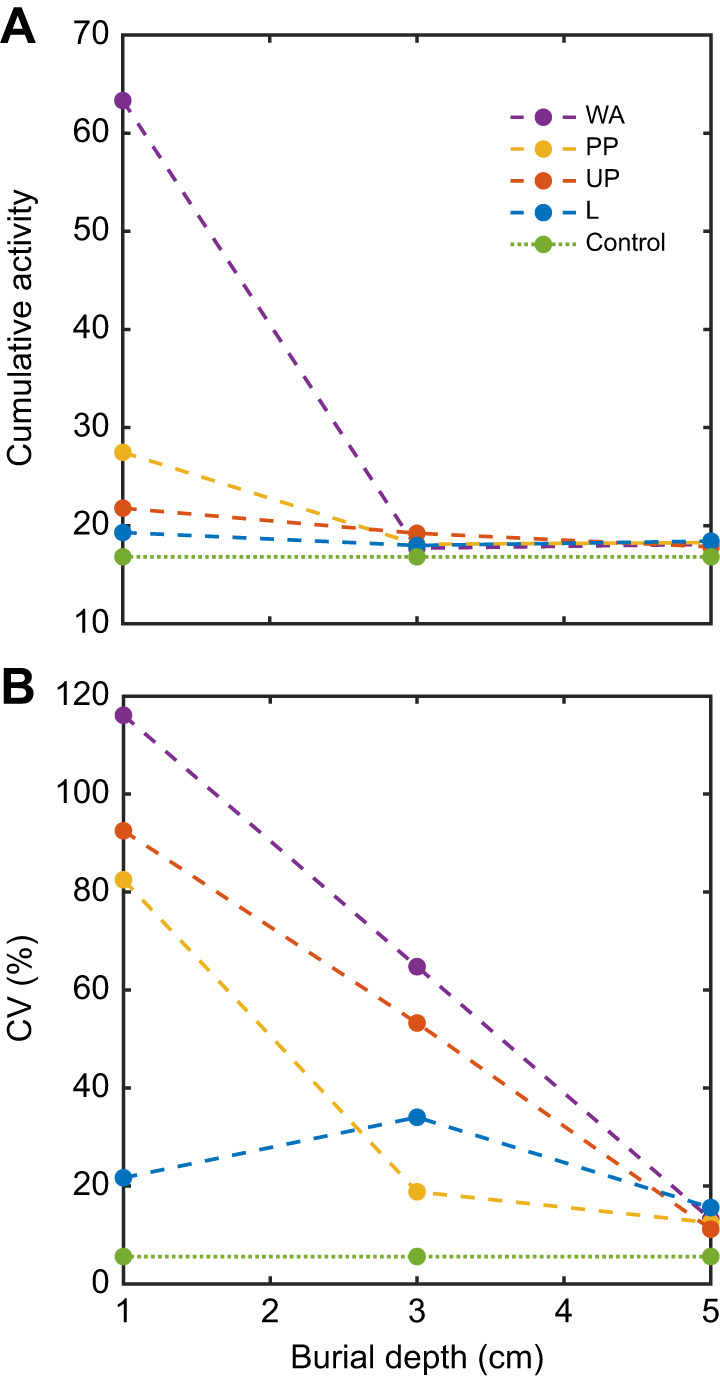
**Activity and variability of fire ants across life stages and burial depths.** (A) Numerical integration (a proxy of cumulative activity) and (B) coefficient of variation (CV; the standard deviation over the mean) for the ⟨|d*I*/d*t*|⟩ sample data provided for fire ants at various life stages (Fig. 3), including larva (L), unpigmented pupa (UP), pigmented pupa (PP) and worker adult (WA), placed under dry glass beads at depths of 1, 3 and 5 cm within a cylindrical container (5 cm diameter and 8 cm length), compared with the control. The lines connecting data points serve to differentiate between distinct phase developments rather than imply trends across untested burial depths.

Interestingly, most curves displayed a decrease in ⟨|d*I*/d*t*|⟩ over time, which we attributed to our protocol: at *t*≈0, the animals were fully buried and likely struggled vigorously for some time. Eventually, they became quiescent with occasional spikes indicating bouts of activity. Mean activity (defined by averaging ⟨|d*I*/d*t*|⟩ over 120 s, reported in Fig. 3 caption) increased with developmental stage and decreased with burial depth, particularly at 1 cm depth. However, at greater burial depths of 3 and 5 cm, it remained relatively consistent. Another approach involves numerically integrating ⟨|d*I*/d*t*|⟩ over time, to gain insight into cumulative activity and total magnitude of the signal (Fig. 4A). This calculation exhibited a similar trend to mean activity. Additionally, the coefficient of variation (CV) of ⟨|d*I*/d*t*|⟩ (the standard deviation over the mean) can assess the relative variability of the signal around the mean (Fig. 4B). We observed that as burial depth increased, CV decreased, indicating a reduction in distant high-magnitude spikes and an increase in frequent low-magnitude spikes. The detection of movement of larval and pupal ants is remarkable given that these stages undergo only minor changes in body posture. The variation of activity with depth can be rationalized by a decrease in activity generated by increased pressure on the animals at greater depths and/or the fact that when the animal is sufficiently outside the laser scattering field, speckle shifts are due to displacements of grains far from the field.

**Fig. 5.**
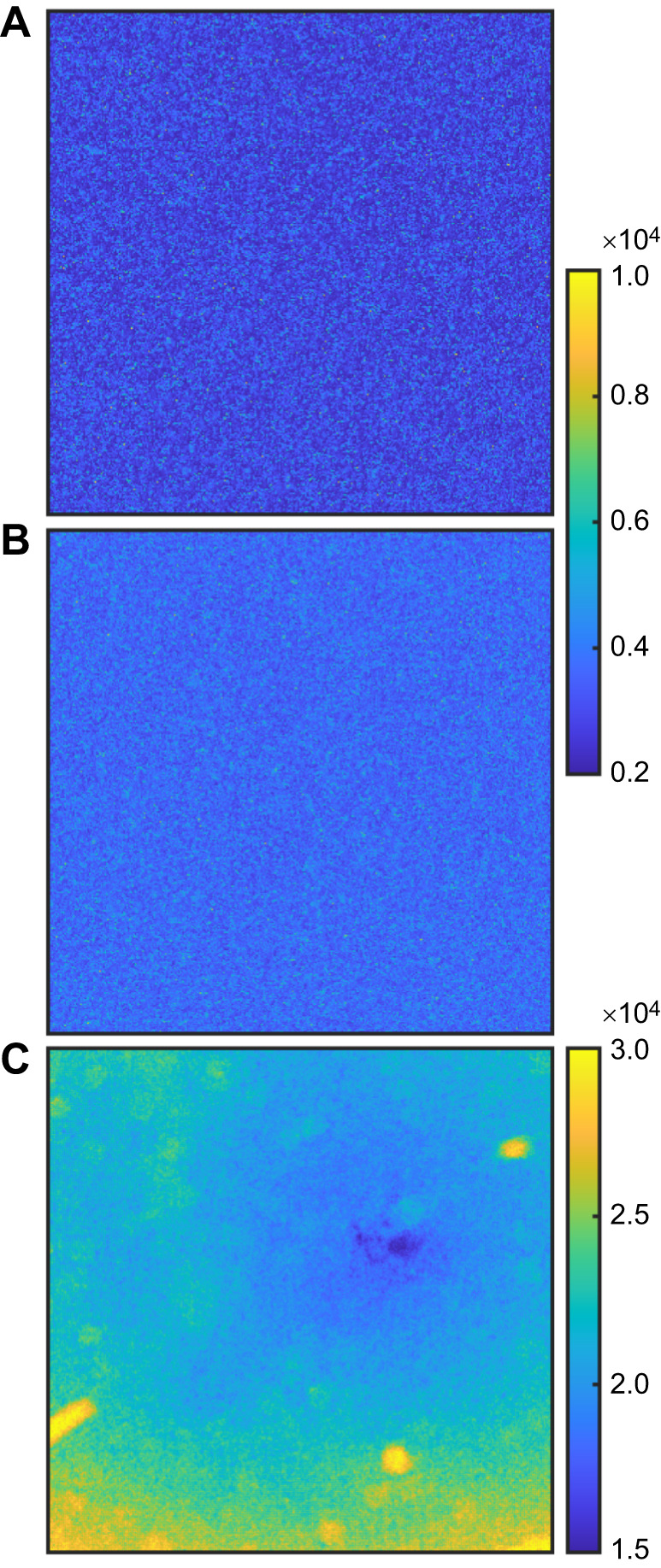
**Resolving spatial activity from speckle patterns.** Color maps for the absolute pixel intensity of the speckle pattern change summed over time (Σ|d*I*/d*t*|) for a 700 × 700 pixel^2^ image under the following conditions: (A) no buried ant (control), or an adult worker ant buried under (B) 1 cm or (C) 0.3 cm of dry glass bead medium within a cylindrical container (5 cm diameter and 8 cm length). The color bar intensity represents variation in pixel intensity, with brighter colors indicating greater changes and dimmer colors signifying lesser changes. The location of the buried ant is close to the center of the image.

Analysis of the time-averaged speckle images did not reveal any distinguishable spatial patterns for adult ants at the experimented burial depths (greater than 1 cm), indicating that the collected light is in the multiple scattering regime (Maret, 1997; Weitz et al., 1993). However spatial information was obtainable at shallow burial depths of 3–4 mm (Fig. 5C), indicating non-diffusive light paths. The shades of blue in Fig. 5C indicate the region of the buried ant. This region exhibited elevated pixel intensity, accompanied by minimal average changes in pixel intensity over time, indicating heightened localized particle movement induced by ant activity. In contrast, the outer perimeter displayed diminished pixel intensity with more pronounced average changes in pixel intensity over time, associated with reduced particle displacement. This resulted in a color map featuring brighter shades of yellow along the outer perimeter and darker hues of blue towards the central region.

Finally, as most natural soils are not fully dry, we also tested the LSI technique for its ability to detect ant activity in wet granular media. The presence of water at concentrations below full saturation (∼0.25–0.3 by volume; Monaenkova et al., 2015) serves to attenuate the light and mechanical agitation, and thus we expected less ability to resolve activity. However, in a complex system like this, where attenuation could result from multiple competing effects, the relative weights of these effects are uncertain. Indeed, obvious detection of ant movement primarily occurred for buried adults at 1 cm depth in the water-saturated medium (Fig. S3), although spikes in activity could be detected in many cases (Fig. S4b).

The approach to discerning ant movement (spikes in activity) involved analyzing instances where the peak magnitudes of ⟨|d*I*/d*t*|⟩ exceeded twice the mean value of the control. This criterion was chosen based on the observation that, in lower developed stages and at greater burial depths, the signal displayed more sparsely spaced spikes. The averaging of these spikes did not yield a significant difference when compared with the control, even though there still existed distinct trains of spikes at greater burial depths. Consequently, the mentioned metric was utilized to evaluate seven trials for each condition for a 2 min backscatter speckle recording using the 3D experimental container. While the majority of ants, across different development stages, exhibited spikes in activity for all tested burial depths within dry glass particles (Fig. S4a), this was not the case in wet media (Fig. S4b). In wet granular media, spikes in activity were primarily observed in later developed stages, specifically worker adult ants. Interestingly, for dry glass particles, there was a slight decrease in spike activity from worker adult ants at depths of 3 and 5 cm. This could potentially be attributed to saturated media hindering particle displacement and absorbing mechanical agitations created by animal movement, leading to animal fatigue and adaption to the surroundings. Preliminary experiments with fully submerged glass particles, featuring a 1 cm water layer above the particle medium, yielded unpromising results with excessive fluctuations in the control experiment (Fig. S5). These fluctuations were possibly due to water currents shifting particles and debris, highlighting limitations of the existing system.

### Conclusion

We tested the ability of the well-known technique of LSI to capture dynamics of animals buried in both dry and wet granular media. Overall, this non-invasive, inexpensive and relatively simple technique demonstrated the ability to detect subtle movements of the target samples in numerous cases. By evaluating the backscattered speckle pattern over time, we captured subtle particle movements induced by animal motion. We used the developmental stages of fire ants to investigate the utility of this technique, finding that the ability to detect ant movement increased over developmental stage. Additionally, the detection of ant movement decreased at greater burial depths and in wet granular media. While the technique thus seems promising for detecting movement in nearly transparent dry granular material, improvements must be made if the technique is to be applied in more diverse (real-world) materials with greater opacity or more complex particle shapes (e.g. the more opaque substrates in Movie 2). Experimentally, one could use higher intensity lasers, different wavelengths and faster cameras. In addition, advances in theory will be needed to interpret the spiking patterns resulting from indirect displacement of grains in the light scattering field originating from localized displacement around the animal (which might not be in the scattering field). Interrogating the diversity of life (Monaenkova et al., 2015; Sharpe et al., 2015b; Gravish et al., 2013) within fully saturated granular media, such as on the ocean bottom (Schmidt-Rhaesa, 2020; Giere, 2009; Du Clos, 2014; Dorgan, 2018; Winter et al., 2012), where laser light can better penetrate the medium (Goldman, 2002), could also be a promising environment in which to use LSI.

## Supplementary Material

10.1242/jexbio.247267_sup1 Supplementary information
