# Supplementary material for: Detecting subtle subterranean movement via laser speckle imaging
Source: J Exp Biol. 2024 Nov 22;227(22):jeb247267. doi: 10.1242/jeb.247267 (PMC11607681; doi:10.1242/jeb.247267)
Supplement: Supplementary information [file jexbio-227-247267-s1.pdf]

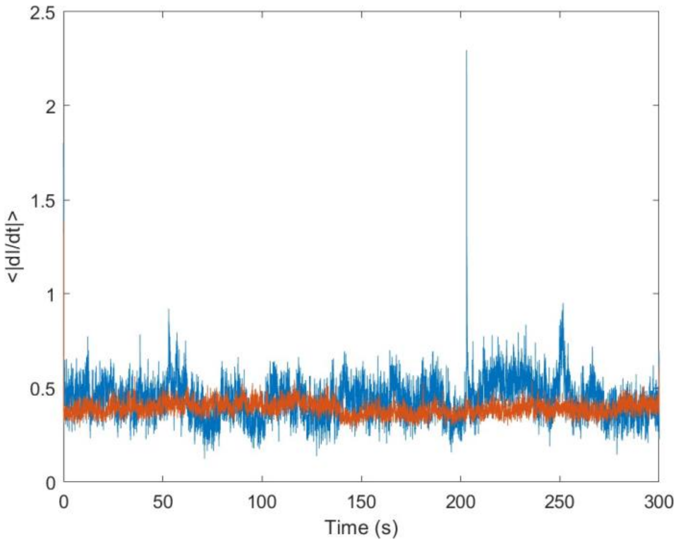

Fig. S1. LSI activity of a fire ant larva at placed under dry glass beads at depths of 1 cm within a within a cylindrical container (5 cm diameter and 8 cm length), using a (low-cost) 532 nm plug-in green laser. In the resulting plots, blue/orange correspond to the presence/absence of a buried animal.

Journal of Experimental Biology • Supplementary information

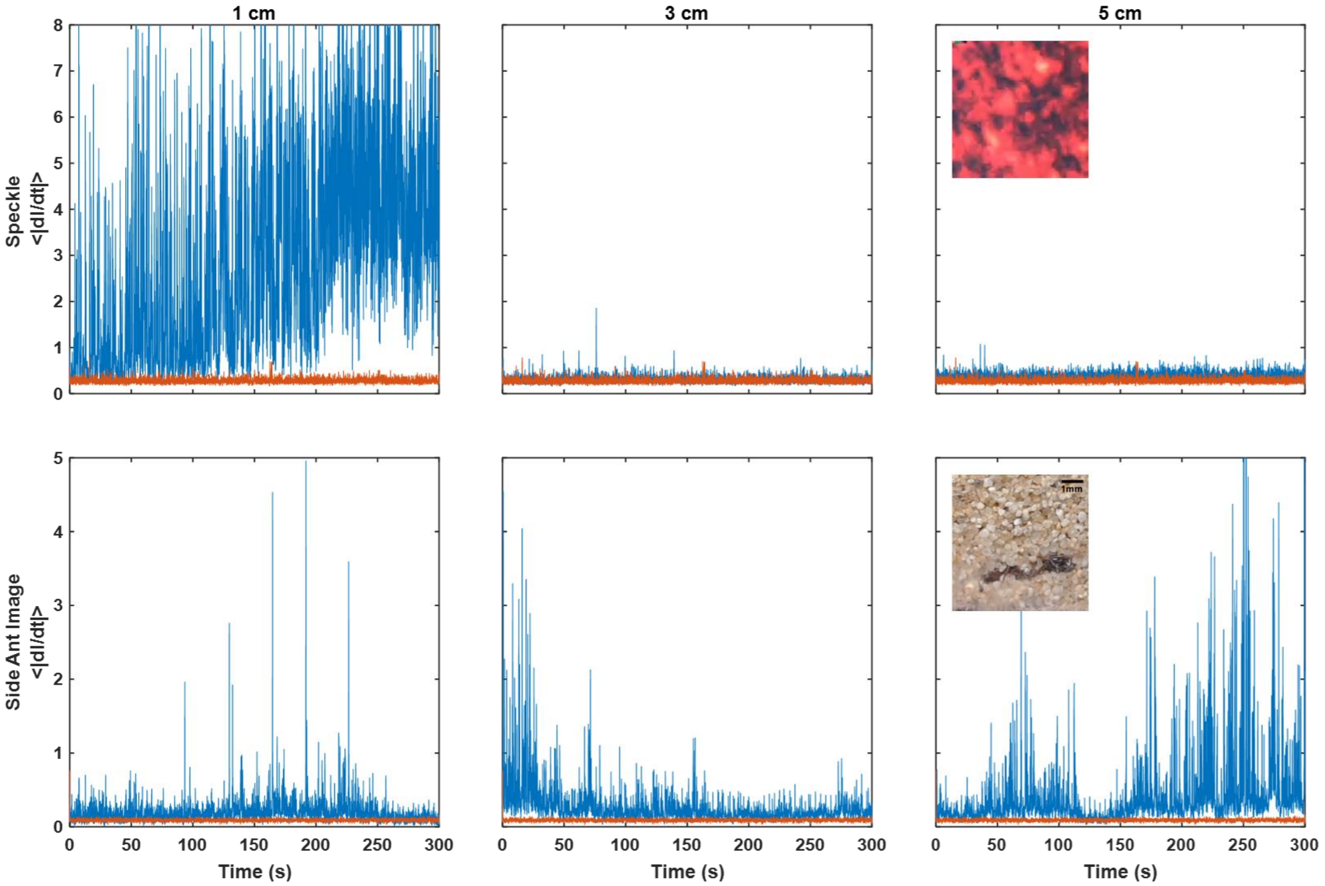

Fig. S2. LSI reveals activity of worker adult fire ant buried under ~500 μm dry natural sand at depths of 1, 3, and 5 cm within a quasi-2D setup (5 cm width, 6 cm height, and 0.5 cm depth). Resulting plots show backscattered speckle pattern and side particle movement for 5-minute recording, with blue/orange correspond to the presence/absence of a buried animal. See Vid. S2 for synchronized video.

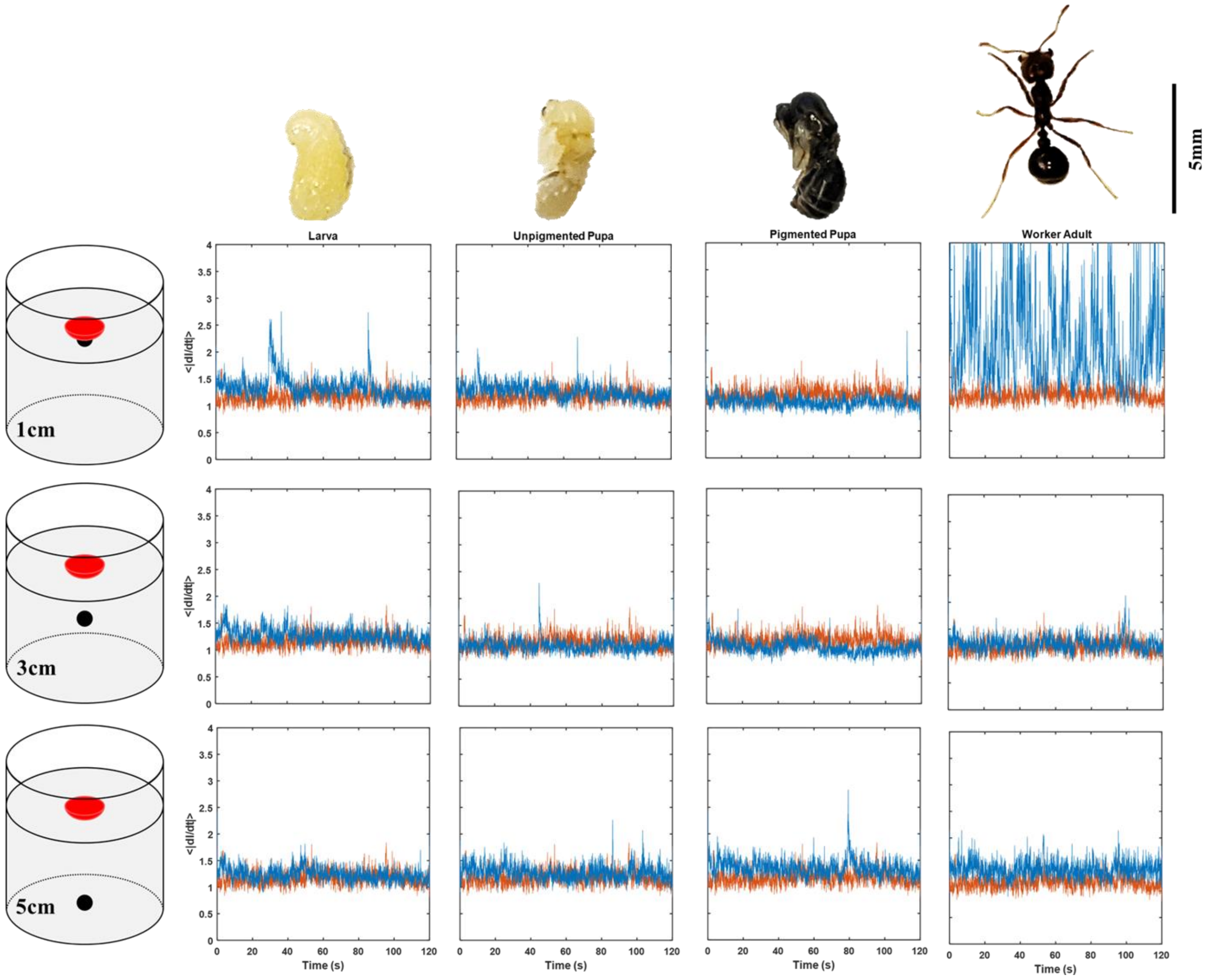

Fig. S3. LSI reveals activity of fire ants at various life stages, including larvae (L), unpigmented pupae (UP), pigmented pupae (PP), and worker adult (WA), placed under 10% saturated glass beads at depths of 1, 3, and 5 cm within a cylindrical container (5 cm diameter and 8 cm length). In the resulting plots, blue/orange correspond to the presence/absence of a buried animal. Mean activity, defined by averaging $< |dI/dt| >$, are as follows: control = 1.17, L_1 = 1.35, L_3 = 1.27, L_5 = 1.24, UP_1 = 1.26, UP_3 = 1.12, UP_5 = 1.27, PP_1 = 1.04, PP_3 = 1.05, PP_5 = 1.36, WA_1 = 2.17, WA_3 = 1.23, WA_5 = 1.41.

Journal of Experimental Biology • Supplementary information

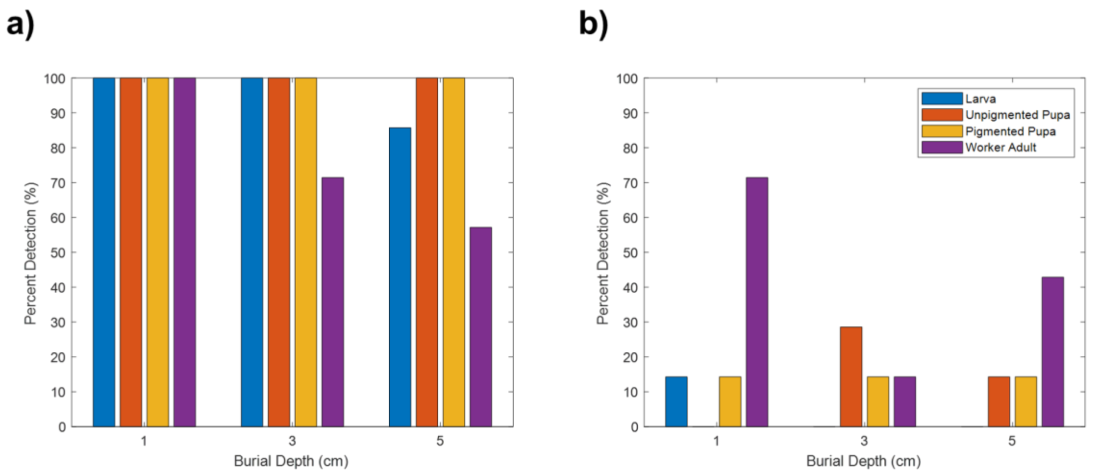

Fig. S4. Percentage of detected ant movement when buried under 1, 3, and 5 cm of (a) dry and (b) 10% water-saturated glass beads out of 7 trials for each development stage of reproductive fire ant (larva, unpigmented pupa, pigmented pupa, and worker adult). The $< |dI/dt| >$ data was analyzed to determine the presence of movement (in comparison to the control) for ants buried under a cylindrical container, with a 2-minute recording of the backscattered speckle pattern. The criteria for discerning ant movement were based on trials where the magnitude peaks of $< |dI/dt| >$ surpassed twice the means value of the control.

Journal of Experimental Biology • Supplementary information

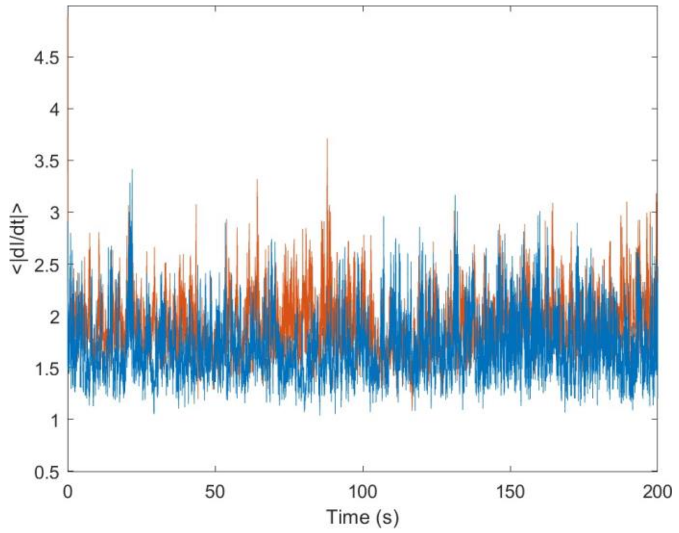

Fig. S5. LSI activity of fire worker adult ant placed under fully submerged glass beads (1 cm layer of water above the glass particle medium) within a quasi-2D setup (5 cm width, 6 cm height, and 0.5 cm depth). In the resulting plots, blue/ orange correspond to the presence/absence of a buried animal.

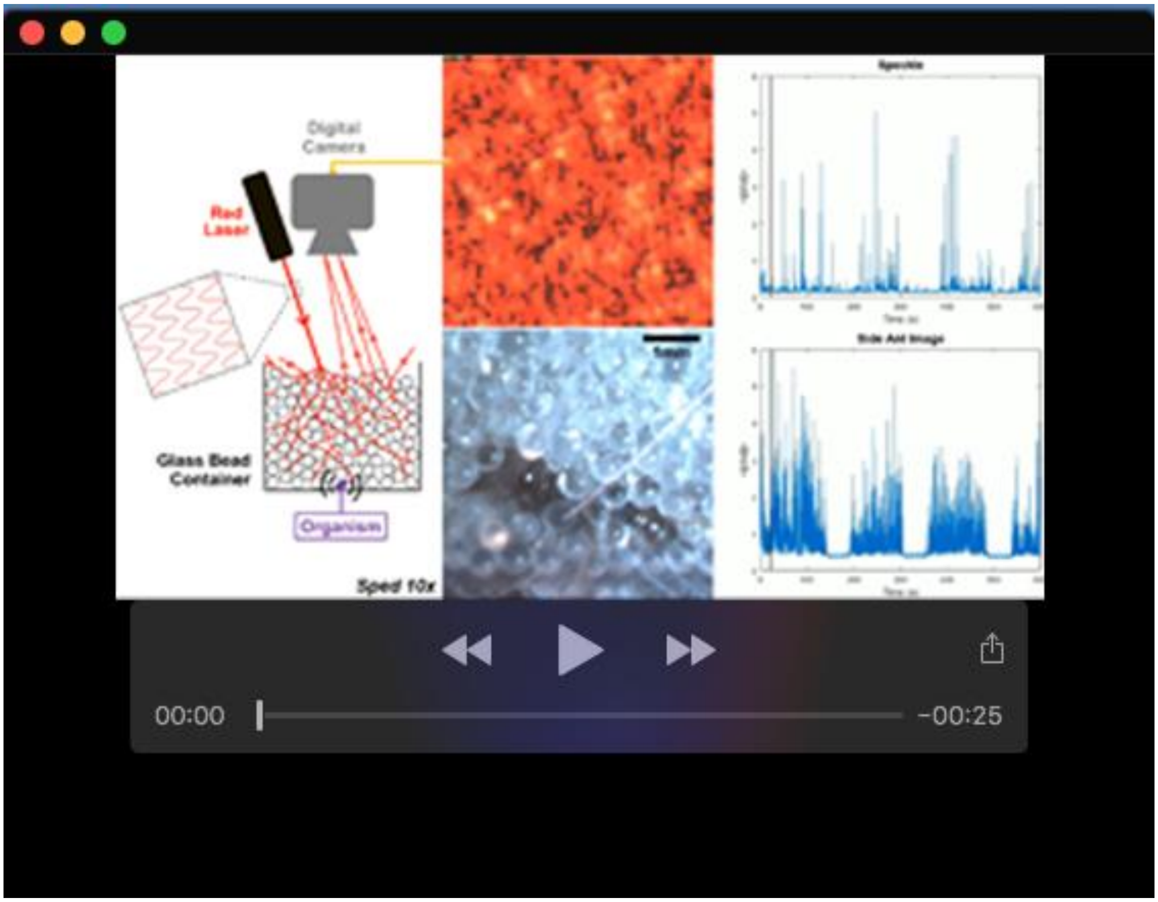

Movie 1. Calibrating the LSI method via a quasi-2D setup: 10-minute recording of the backscattered speckle pattern and side particle movement induced by a worker ant buried under 5 cm of dry glass beads in a quasi-2D container (5 x 6 x 0.5 cm^3). Plots depict mean absolute pixel intensity change over time, detonated as $< |dI/dt| >$.

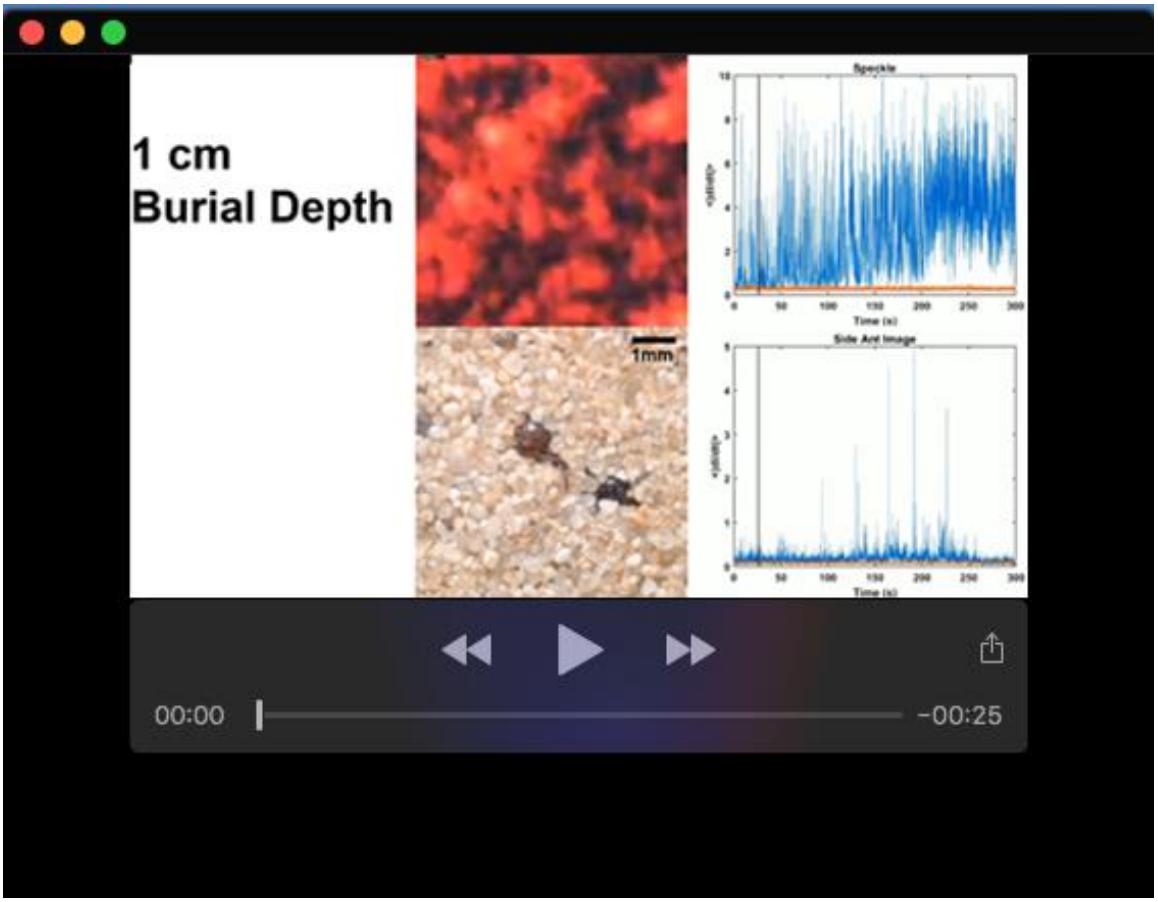

Movie 2. Demonstrating the LSI method using natural sand within a quasi-2D setup: 5-minute recording of the backscattered speckle pattern and side particle movement induced by a worker adult ant buried under 1, 3, and 5 cm of dry sand in a quasi-2D container (5 x 6 x 0.5 cm^3). Plots depict mean absolute pixel intensity change over time, detonated as <|*dI/dt*|>, with blue/orange correspond to the presence/absence of the buried ant.

Journal of Experimental Biology • Supplementary information
